# Murine Missing in Metastasis (MIM) Mediates Cell Polarity and Regulates the Motility Response to Growth Factors

**DOI:** 10.1371/journal.pone.0020845

**Published:** 2011-06-09

**Authors:** Dan Yu, Xiaoguo H. Zhan, Shuqiong Niu, Irina Mikhailenko, Dudley K. Strickland, Jianwei Zhu, Meng Cao, Xi Zhan

**Affiliations:** 1 The Center for Vascular and Inflammatory Diseases, University of Maryland School of Medicine, Baltimore, Maryland, United of States of America; 2 Department of Physiology, University of Maryland School of Medicine, Baltimore, Maryland, United of States of America; 3 Department of Surgery, University of Maryland School of Medicine, Baltimore, Maryland, United of States of America; 4 Department of General Surgery, The Affiliated Hospital, Nantong University, The People's Republic of China; 5 Department of Pathology, University of Maryland School of Medicine, Baltimore, Maryland, United of States of America; 6 University of Maryland Marlene Stewart Greenebaum Cancer Center, University of Maryland School of Medicine, Baltimore, Maryland, United of States of America; Cardiff University, United Kingdom

## Abstract

**Background:**

Missing in metastasis (MIM) is a member of the inverse BAR-domain protein family, and in vitro studies have implied MIM plays a role in deforming membrane curvature into filopodia-like protrusions and cell dynamics. Yet, the physiological role of the endogenous MIM in mammalian cells remains undefined.

**Principal Findings:**

We have examined mouse embryonic fibroblasts (MEFs) derived from mice in which the MIM locus was targeted by a gene trapping vector. MIM^−/−^ MEFs showed a less polarized architecture characterized by smooth edges and fewer cell protrusions as compared to wild type cells, although the formation of filopodia-like microprotrusions appeared to be normal. Immunofluorescent staining further revealed that MIM^−/−^ cells were partially impaired in the assembly of stress fibers and focal adhesions but were enriched with transverse actin filaments at the periphery. Poor assembly of stress fibers was apparently correlated with attenuation of the activity of Rho GTPases and partially relieved upon overexpressing of Myc-RhoA^Q63L^, a constitutively activated RhoA mutant. MIM^−/−^ cells were also spread less effectively than wild type cells during attachment to dishes and substratum. Upon treatment with PDGF MIM^−/−^ cells developed more prominent dorsal ruffles along with increased Rac1 activity. Compared to wild type cells, MIM^−/−^ cells had a slower motility in the presence of a low percentage of serum-containing medium but migrated normally upon adding growth factors such as 10% serum, PDGF or EGF. MIM^−/−^ cells were also partially impaired in the internalization of transferrin, fluorescent dyes, foreign DNAs and PDGF receptor alpha. On the other hand, the level of tyrosine phosphorylation of PDGF receptors was more elevated in MIM depleted cells than wild type cells upon PDGF treatment.

**Conclusions:**

Our data suggests that endogenous MIM protein regulates globally the cell architecture and endocytosis that ultimately influence a variety of cellular behaviors, including cell polarity, motility, receptor signaling and membrane ruffling.

## Introduction

MIM was initially recognized as a putative metastasis suppressor due to its frequent silence in a subset of metastatic cells, including breast, bladder, prostate and stomach cancers [1]–[5]. The gene of MIM encodes a protein product that contains a C-terminal WASP-homology 2 (WH2) motif for binding to monomeric actin and an N-terminal region of 250 amino acids that forms a homodimer. This dimmerized N-terminal motif is structurally similar to that of insulin receptor substrate protein 53 (IRSp53), and distantly related to Bin-Amphiphysin-Rvs (BAR) domains, which are present in a series of membrane modeling or BAR-domain proteins [6], [7]. Unlike conventional BAR-domains, which usually take a crescent shape with the membrane interacting surface on the concave face, the BAR domain of MIM and IRSp53 has a zeppelin profile with the membrane binding surface on the convex face [8]–[10], and thereby defining a distinct subfamily of the BAR-domain proteins as the inverse BAR (I-BAR) domain family. In line with this structural feature, recombinant I-BAR domain peptides have a high affinity for PI(4,5)P_2_-enriched membranes [11] and induce membrane protrusions in an opposite direction to that induced by conventional BAR-domain peptides [12]. Similarly, overexpression of the I-BAR proteins often results in over-extruded membrane protrusions that are morphologically similar to filopodia [13], [14]. Thus, I-BAR proteins have been thought to participate in the deformation of membrane curvature into filopodia-like protrusions, which may be relevant to cellular dynamics such as cell migration, invasion and endocytosis [8], [15].

There is emerging evidence for the implication of MIM in various signaling pathways to regulate the actin cytoskeleton reorganization. The MIM I-BAR domain binds to and cross-links filamentous (F) actin in vitro [16], and directly associates with Rac1 [17]. The WH2 domain of MIM has a high affinity for GTP–bound actin monomer [11], [18]. Furthermore, ectopically expressed MIM protein co-localizes and directly associates with cortactin, an Arp2/3 binding protein during actin polymerization, and promotes the cortactin-mediated actin assembly in vitro [19]. In *Drosophila*, a mutant with depletion of the MIM locus exhibits a defect in the directional migration of border cells by inhibiting endocytosis and antagonizing the activities of the CD2-associated protein/cortactin complex in these cells [20]. MIM is also subjected to regulations by extracellular stimuli. Upon PDGF treatment MIM undergoes tyrosine phosphorylation in a Src-dependent manner [21]. Expression of MIM in mesenchymal cells is upregulated by Sonic Hedgehog (Shh) [16], and is implicated in cilia maintenance, Shh responsiveness, and de novo hair follicle formation [22]. In spite of these reports, our current knowledge about the function of MIM in mammalian cells was largely based on studies using MIM transfected or overexpressing cells. Thus, the physiological role of the endogenous MIM in cell dynamics, in particularly in mammalian species, remains to be defined.

In this study we have characterized embryonic fibroblasts derived from the mice in which the MIM gene was targeted by a gene trapping vector. Although we did not observe a significant defect in the formation of filopodia, MIM^−/−^ cells displayed marked alterations in the actin cytoskeletal architecture, motility response to extracellular cures, and internalization of transferrin, fluorescent dyes and PDGF receptors. Our data suggests that MIM regulates globally the cellular architecture that influences a variety of cellular properties.

## Results

### 1. Generation of MIM knockout mice

**Figure 1:**
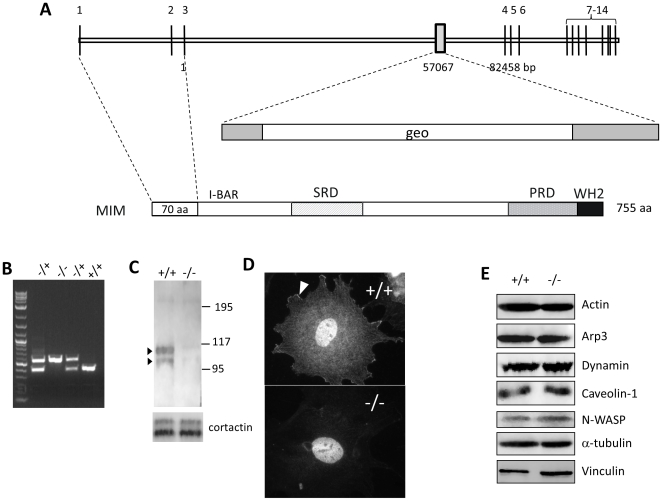
Preparation of MIM knockout mice. (A) schematic illustration of the genomic structure of the murine MIM gene. The trapping vector, which carries a gal-neo (geo) fusion gene, targeted at the position corresponding to the nucleotide 57,067 in the third intron. (B) Genotyping of MIM knockout mice by PCR with the primers flanking the trapping vector. The top band (625 bp) indicates the mutant allele, and the lower band (400 bp) indicates the wild-type allele. (C) Western blot analysis of MEFs using MIM antibody, which detected a MIM characteristic duplex of p115 and p100 in the lysate of the MIM^+/+^ cells. The same membrane was re-blotted with cortactin antibody for the loading control (the lower panel). (D) Immunostaining of a MEF cell using MIM antibody. The arrow head indicates a representative lamellipodia staining. The nuclear staining was likely non-specific and observed with both MIM^−/−^ and MIM^+/+^ cells. (E) Expression of several cortical proteins in MIM^+/+^ and MIM^−/−^ MEFs.

To obtain insight into the physiological function of MIM, we have prepared MIM knockout mice by utilizing an embryonic stem (ES) cell line carrying a gene trapping vector (pGT0lxf) at the locus between the 3^rd^ and the 4^th^ exons (Fig. 1A). Microinjection of ES cells into blastocysts and subsequent transfer into a pseudo-pregnant recipient mouse resulted in two chimeras, both of which were able to transmit ES cells into the germ cells and were further used to generate heterozygous and homozygous mice. MIM knockout mice were further backcrossed with C57BL/6 mice for more than six generations to generate a nearly homogenous genetic background. The presence of the mutated MIM alleles in heterozygous and homozygous mice was confirmed by genomic PCR (Fig. 1B) and by immunohistochemical staining of the cerebellum in which MIM is highly expressed [1] (Fig. S1).

MIM^−/−^ mice developed normally and were fertile, indicative of a dispensable role of MIM in embryonic development. To determine whether depletion of MIM could impact on cellular behaviors in vitro, we examined MEFs derived from MIM^+/+^ and MIM^−/−^ mice. Western blot of MIM^+/+^ MEFs using a polyclonal MIM antibody detected a duplex around the positions of 105/110 kDa, respectively (Fig. 1C). Staining with the MIM antibody revealed a diffused immunoreactivity in the cytoplasm and at the leading edge of MIM^+/+^ but not MIM^−/−^ cells (Fig. 1D), the pattern that was similar to that of ectopically expressed GFP-MIM protein in NIH3T3 cells [21]. A heavy but likely non-specific nuclear staining was also observed in both MIM^+/+^ and MIM^−/−^ cells. Because MIM protein is often associated with the actin-enriched cellular cortex, we further analyzed a series of cortical and cytoskeletal proteins including actin, dynamin, caveolin-1, N-WASP, alpha-tubulin and vinculin, and found no significant difference in the expression of these proteins between MIM^−/−^ and wild type cells (Fig. 1E).

### 2. MIM^−/−^ cells display reduced polarity and an altered actin cytoskeletal architecture

**Figure 2:**
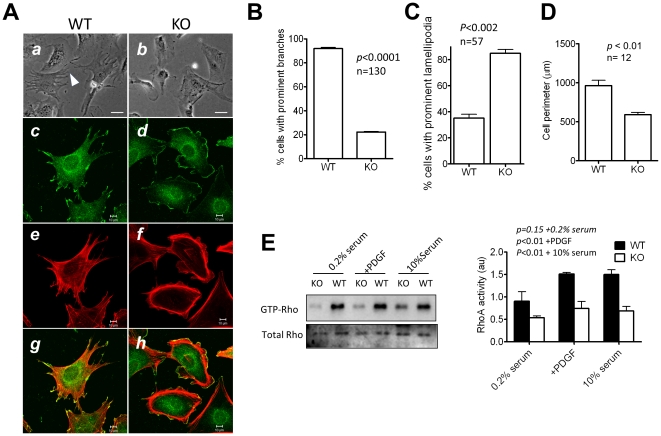
MIM^**−/−**^ cells display a distinct morphology and altered actin cytoskeleton reorganization. (A) MIM^+/+^ (WT) MEFs (*a*, *c*, *e*, and *g*) and MIM^−/−^ (KO) cells (*b*, *d*, *f* and *h*) were grown in 10% serum-containing medium on fibronectin covered slips, inspected by phase-contrast view (*a* and *b*) or by confocal microscopy following co-staining with cortactin antibody (*c* and *d*) and phalloidin (*e* and *f*). The images *c* and *e* were merged as *g*, and the images *d* and *f* were merged as *h*. Scale bars: *a* and *b*, 50 µm; *c–h*, 10 µm. (B) Quantification of the percentage of cells bearing prominent body projections as revealed under phase-contrast microscopy. (C) Quantification of the cell surface perimeter measured by MetaMorph program. (D) Quantification of the percentage of cells with prominent cortactin-enriched lamellipodia. (E) Activation of Rho is elevated in MIM^−/−^ cells. Cells were arrested in 0.2% serum-containing medium and stimulated with 30 ng/ml PDGF for 10 min. The level of GTP-Rho was measured by pull-down assay as described in the Materials and Methods. As a control, cells grown in 10% serum were also analyzed in parallel. Quantification of Rho activation is presented on the right based on three experiments (mean ± SEM). All the *p* values (*t*-test) refer to the difference between MIM^+/+^ and MIM^−/−^ cells.

MIM^−/−^ cells proliferated similarly as MIM^+/+^ cells *in vitro* (Fig. S2). However, they were distinguished from MIM^+/+^ cells, most of which showed a highly polarized shape characterized by forming long and actin-enriched projections (Fig. 2A-*a*). In contrast, the majority of MIM^−/−^ cells displayed rather smooth edges (Fig. 2A-*b*) with few exhibiting projections at variant degrees (Fig. 2B). The difference between wild type and MIM^−/−^ cells was more evident after staining with antibody against cortactin, a lamellipodia associated protein. More than 80% of MIM^−/−^ cells showed smooth edges outlined by cortactin-enriched lamellipodia (Fig. 2A-*d*, 2C), while the cortactin staining was mainly associated with polarized membrane ruffles at tips of the projections in wild type cells (Fig. 2A-c). MIM^−/−^ cells also tended to display a smaller cellular body with an average of surface perimeter 30% less than that of MIM^+/+^ cells (Fig. 2D). Phalloidin staining for filamentous actin further revealed altered actin cytoskeleton networks within MIM^−/−^ cells. Whereas wild type cells were rich in ventral stress fibers that were aligned in the direction of cell projections (Fig. 2A-*e*), these straight fibers were much less evident in MIM^−/−^ cells (Fig. 2A-*f*). Instead, MIM-deficient cells were enriched with transverse or arc-like stress fibers that outlined the cells. Staining with vinculin antibody also revealed that MIM^−/−^ cells developed numerous small focal adhesions at the peripheral but fewer adhesion sites along with stress fibers (Fig. S3). When maintained in 10% serum-containing medium, MIM^−/−^ cells exhibited more dynamic leading edges than wild type MEFs (Video S1 and S2).

To further evaluate the effect of MIM deficiency on stress fibers, we measured the activation of Rho proteins, which are known to play a key role in the formation of stress fibers [23]. As shown in Fig. 2E, the level of activated Rho (GTP-Rho) in MIM^−/−^ cells was significantly less than that in wild type cells under the conditions of either 10% serum-containing medium or 0.2% serum medium supplemented with and without stimulation with PDGF. The reduced Rho activity was unlikely due to a possible alteration in the expression of Rho proteins because MIM^−/−^ cells had a normal Rho expression either in the presence of serum or stimulated by PDGF (Fig. S4). Transient transfection with an activated RhoA (myc-RhoA^Q63L^), but not a wild type RhoA (GFP-RhoA), partially restored the stress fiber formation and the polarity of MIM^−/−^ cells (Fig. S5), suggesting that the diminished Rho activity was partially responsible for the reduced assembly of stress fibers in MIM^−/−^ cells.

Significant morphological changes were also found with MEFs derived from different MIM^−/−^ mice (data not shown) or established MIM^−/−^ cell lines (Fig. S6). To further verify that the phenotype was truly due to the lack of the function of MIM, we had attempted to re-introduce a GFP or Myc-tagged MIM construct into MIM^−/−^ cells by either DNA-mediated transfection or retrovirus infection. Both the efforts had yielded poor efficiency in introduction of MIM constructs into MIM^−/−^ cells (Fig. S7A). Transfection with a different gene such as GFP-cortactin was also poorer in MIM KO cells than wild type cells (Fig. S7B), suggesting that depletion of MIM expression inhibited the uptake of foreign DNAs, though the degree of the inhibition appeared to vary depending upon the nature of DNAs. Although the extremely poor efficiency of transfection of MIM constructs had made it difficult to evaluate extensively their ability to rescue MIM deficiency, in those few GFP-MIM transfected cells, prolonged membrane projections and extremely polarized cell bodies were evident (Fig. S7C), confirming the function of MIM in cell shape changes.

We also examined a possible effect of MIM deficiency on the formation of filapodoia. Under the condition either without (data not shown) or with growth factors (Fig. S8A and 8B), we observed neither apparent difference in filopodia formation between MIM^−/−^ and MIM^+/+^ fibroblasts (*p>0.8*) (Fig. S8C) nor significant increase in the activation of Cdc42 (Fig. S8D), a key signaling molecule that regulates filopodia formation. Therefore, we conclude that MIM is dispensable for filopodia assembly in MEFs. However, we could not rule out a redundant function of other I-BAR domain proteins in filopodia formation.

### 3. MIM^−/−^ cells were impaired in cell spreading during attachment

**Figure 3:**
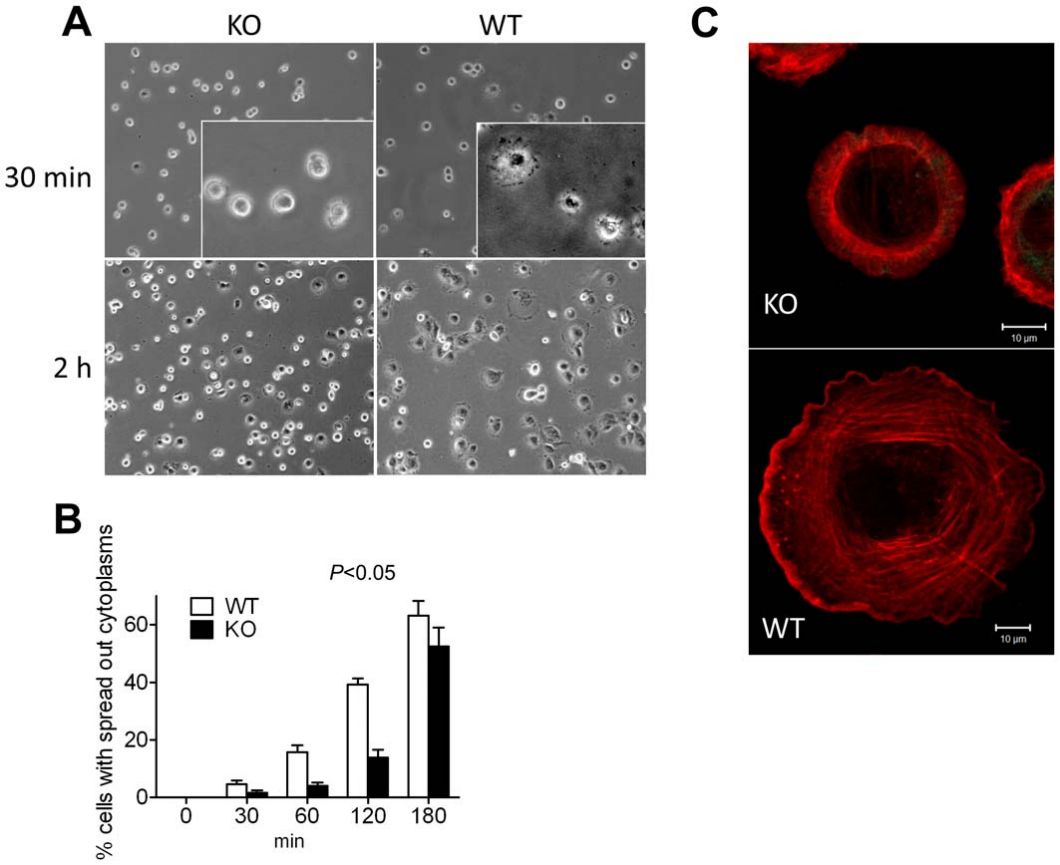
MIM^**−/−**^ MEFs were impaired in spreading during cell attachment. (A) Cells were trypsinized, plated on fibronectin-coated coverslips in a serum-containing medium, fixed at 30 min and 2 h and inspected by phase-contrast microscopy. An enlarged area in each 30-min image was shown in inset. (B) The number of cells with apparent cytoplasm extensions was countered at different times after plating. The data represents mean ± SEM (n = 3). The *p* value (Anova test) refers to the difference between MIM^−/−^ and MIM^+/+^ cells during the course of attachment. (C) Cells at 2 h after plating were fixed, stained with phalloidin and scanned with confocal microscope with each optical section of 0.30 µm in thickness. The images corresponding to different sections were compiled by Zeiss LSM Image browser.

We were also interested in whether MIM deficiency would affect cell attachment. As shown in Fig. 3A, within 30 min after seeding wild type cells had started to spread out by showing extended cytoplasm, while most attached MIM^−/−^ cells remained rounded up (Fig. 3A, insets). After 2 h when 40% of the MIM^+/+^ cells displayed extensively extended cytoplasm, only 15% of the MIM^−/−^ cells did so (Fig. 3B). By 3 h, the difference between wild type and MIM^−/−^ cells was less evident. Confocal microscopy further revealed that many MIM^−/−^ cells developed a smooth edge during spreading (Fig. 3C). In contrast, wild type cells tended to develop a waved leading edge.

### 4. MIM^−/−^ cells showed increase in membrane dynamics in response to PDGF

**Figure 4:**
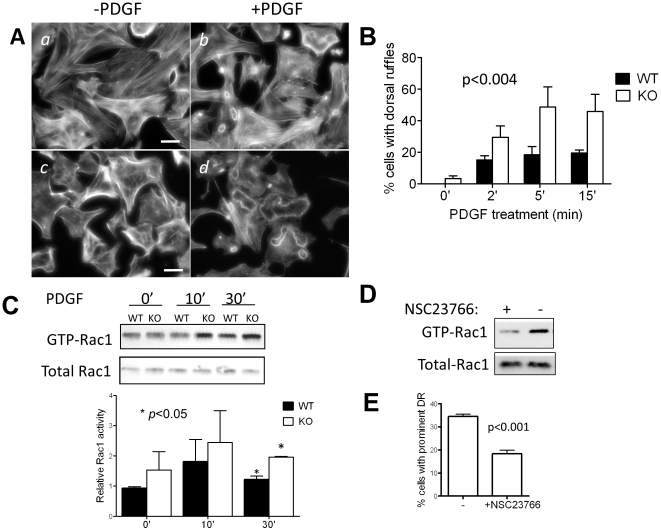
PDGF induces prominent dorsal ruffles in MIM^**−/−**^ MEFs. (A) MIM^+/+^ (*a* and *b*) and MIM^−/−^ (*c* and *d*) cells were arrested by incubating for 24 h in 0.2% serum-containing medium and then treated with PDGF for 10 min (*b* and *d*) followed by staining with phalloidin. The stained cells were inspected by epifluorescent microscopy. Scale bar: 50 µm. (B) Quantification of dorsal ruffles. Cells were treated with PDGF for the times as indicated. The number of cells showing large ruffling areas was counted. The data shown are the mean ± SEM based on four independent experiments. In each experiment 70 cells were analyzed. The *p* value was calculated by Anova test, referring to the difference between MIM^−/−^ and MIM^+/+^ MEFs during the response to PDGF. (C) PDGF treated cells were analyzed for Rac1 activation by pull-down assay followed by Western blot using anti-Rac1 antibody. The Rac1 activation was quantified based on three independent experiments. (D) Quiescent MIM^−/−^ cells grown on fibronectin-coated coverslips were treated with and without Rac1 inhibitor NSC23766 at the concentration of 50 µM for 48 h. The treated cells were then stimulated with PDGF for 10 min, and the Rac1 activation was measured as above. (E) NSC23766 treated cells were also treated with PDGF, and the formation of dorsal ruffles (DR) was quantified based on three independent experiments.

The role of MIM in the actin cytoskeleton and morphogenesis was further analyzed when cells were stimulated by PDGF, a growth factor that often induces dorsal ruffles on fibroblasts [24]. Both MIM^+/+^ and MIM^−/−^ MEFs showed the formation of dorsal ruffles upon PDGF treatment (Fig. 4A). However, those on MIM^−/−^ cells were more prominent than MIM^+/+^ cells (Fig. 4A, *d* v *b*), and the number of MIM^−/−^ cells developing extensive dorsal ruffles was significantly higher than that of MIM^+/+^ cells during the course of PDGF stimulation (*p<0.004*) (Fig. 4B). At 5 min more than two-fold difference between MIM^−/−^ and MIM^+/+^ cells was observed. We also detected higher contents of GTP-Rac1 in MIM^−/−^ cells than those of MIM^+/+^ cells after 10 and 30 min of PDGF treatment (Fig. 4C) or during early responses to PDGF (Fig. S9). To verify the necessity of Rac activation in membrane ruffling, MIM^−/−^ cells were treated with NSC23766, a Rac selective inhibitor [25]. The drug treatment reduced the PDGF-mediated Rac1 activation in MIM^−/−^ cells by nearly 50% (Fig. 4D), which was apparently correlated with 50% deduction in dorsal ruffling (Fig. 4E), implying a role of Rac activation in the dorsal ruffling mediated by MIM deficiency.

### 5. MIM regulates cell motility in response to growth factors

**Figure 5:**
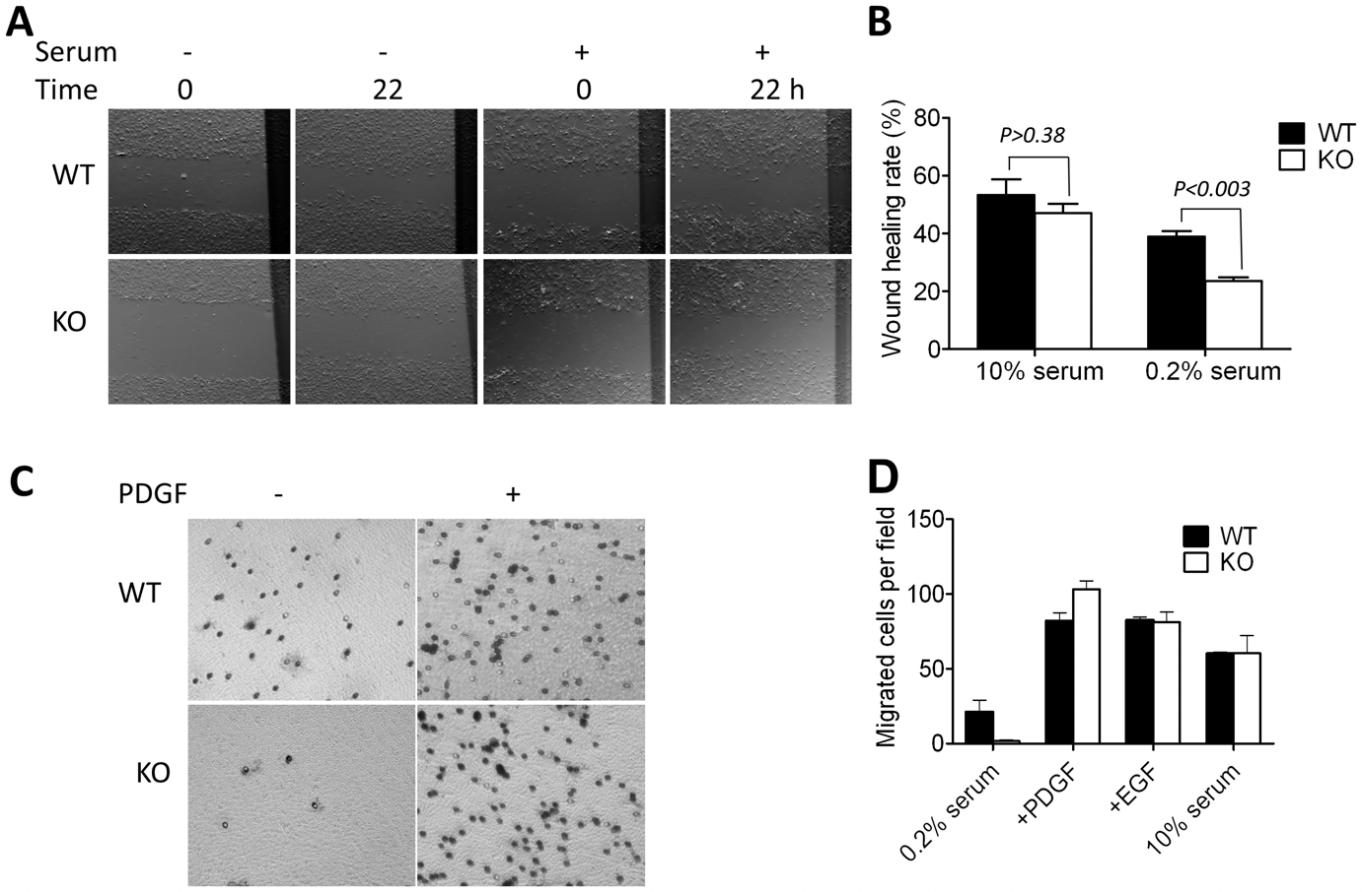
MIM deficiency affects cell motility. (A) Cells were grown in 10% serum-containing medium until confluence, then wounded by rubber policeman and incubated in medium containing either 10% or 0.2% of serum. The images showing the same wounded areas were taken at the beginning of incubation (0) and 22 h later. (B) Wound-healing rates were measured as described in the Materials and Methods. (C) Cells were arrested in 0.2% serum-containing medium and placed on the top chamber of Transwell plates in which the bottom chamber was filled with 0.2% serum medium or medium containing PDGF (30 ng/ml). The plates were incubated for 4 h, stained and photographed. (D) Quantification of the motility of cells in Transwell plates containing 0.2% serum, 30 ng/ml PDGF, 30 ng/ml EGF and 10% serum, respectively. All the presented values are the mean ± SEM of three independent experiments.

We next investigated the effect of MIM deficiency on cell migration, the property that is altered by either overexpression or suppression of MIM as reported previously [4], [19]. The cell motility was first evaluated using a monolayer wound healing assay under the conditions where cells were incubated in either 0.2% or 10% serum-containing medium. In 0.2% serum medium, MIM^−/−^ cells migrated 40% slower than MIM^+/+^ cells (Fig. 5A and 5B). However, in the presence of 10% serum the motility of MIM^−/−^ cells increased to a degree similar to that of MIM^+/+^ cells (*p*>0.38). The increased cell motility in the presence of 10%-serum was unlikely due to a possible increase in cell populations because MIM^−/−^ MEFs showed a similar growth rate as wild type cells under the same condition (Fig. S2). The effect of MIM deficiency on cell motility was also evaluated by Transwell migration assay. When incubated in 0.2% serum-containing medium, MIM^−/−^ cells were nearly immotile and migrated at a rate only 10% of that of MIM^+/+^ cells (Fig. 5C). Upon adding of PDGF or EGF or 10% serum to the lower chamber of Transwell plates, the motility of MIM^−/−^ cells was nearly restored (Fig. 5D). Thus, MIM regulates cellular movement in a manner depending upon extracellular stimuli.

### 6. MIM is positively implicated in the uptake of extracelluar particles

**Figure 6:**
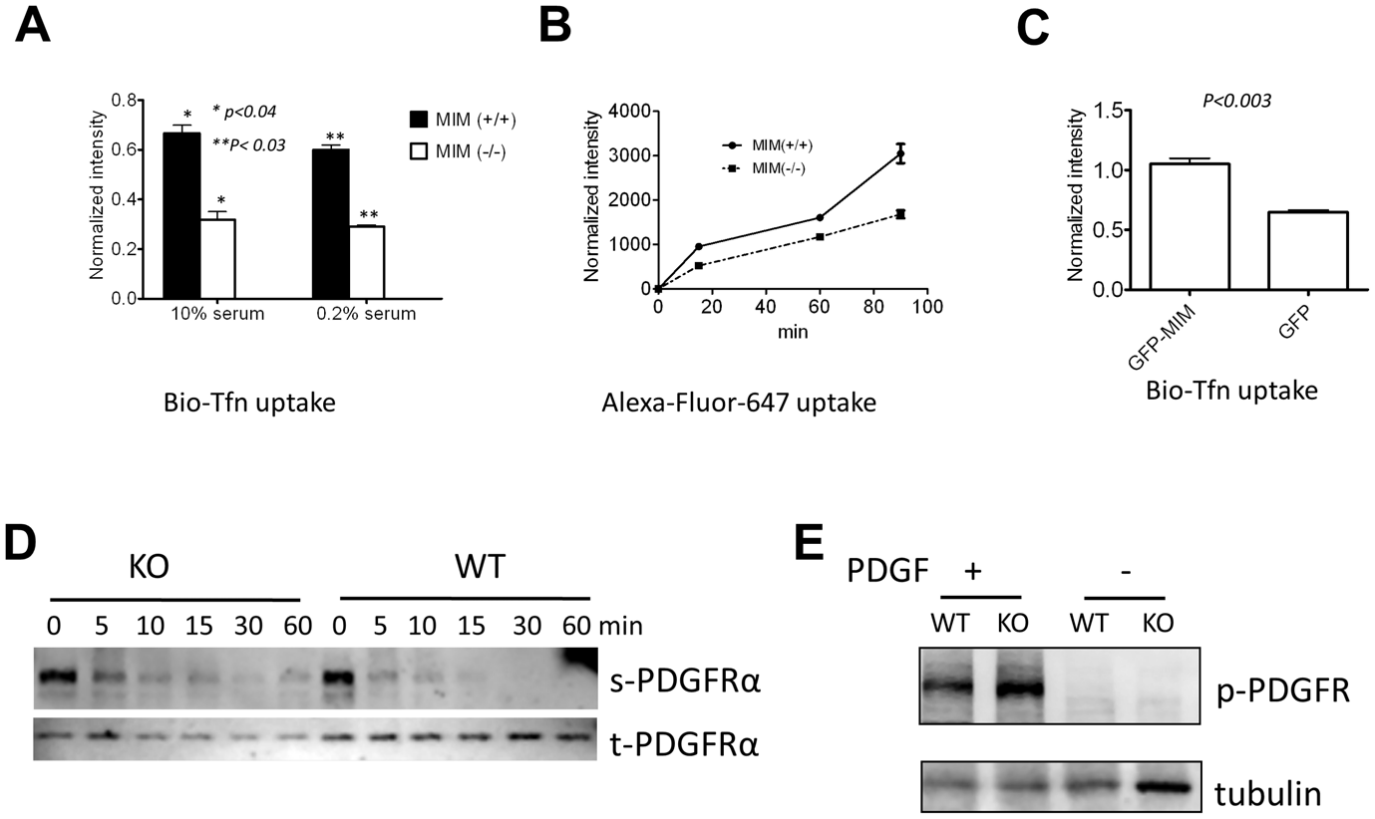
MIM plays a positive role in internalization of extracellular particles. (A) MEFs were grown in medium supplemented with either 0.2% or 10% serum and incubated with biotin-labeled transferrin (Bio-Tfn) for 5 min. Uptake of Bio-Tfn was measured as described in the Materials and Methods. (B) Cells were treated with Alexa Fluor 647 and incubated for the times as indicated. Internalization of the fluorescent dye was measured by flow cytometry. (C) NIH3T3 cells infected by retroviruses encoding GFP-MIM or GFP only were incubated with Bio-Tfn for 5 min. The internalized Bio-Tfn was measured as described in (A). All the error bars represent the mean ± SEM of three independent experiments. P values (*t* test) refer to the difference between the samples as indicated. (D) Quiescent MEFs were stimulated with PDGF (50 ng/ml) for the times as indicated. The cell surface (s) and total (t) PDGFRα proteins were analyzed as described in the Materials and Methods. The data represents two-independent experiments. (E) Quiescent MEFs were stimulated with PDGF for 5 min. The total cell lysates were subjected to Western blot using anti-phosphotyrosine antibody (4G10).

To analyze the role of MIM in the internalization of extracellular particles, we analyzed the uptake of biotin-labeled transferrin under the conditions when cells were grown in either 10% or 0.2% serum-containing medium. Under either of the conditions, MIM^−/−^ cells showed an over 50% decrease in the ability to internalize transferrin (Fig. 6A). A similar decrease was also observed in the internalization of fluorescent dye Alexa Fluor 647 (Fig. 6B), which was driven by micropinocytosis. To confirm the implication of MIM in endocytosis, we further analyzed the uptake of transferrin with MIM overexpressors [21] and observed about 40% increase in the endocytosis in NIH3T3 cells overexpressing GFP-MIM as compared to the cells expressing GFP alone (Fig. 6C). We also analyzed endocytosis of PDGF receptor alpha form (PDGFRα) in MEFs. Although PDGFRα was rapidly internalized in both wild-type and MIM^−/−^ cells upon stimulation with PDGF, significant amounts of surface receptors remained after 30 min in MIM^−/−^ cells (Fig. 6D). Tyrosine phosphorylation analysis further revealed a higher level of phosphorylated PDGF receptors in MIM^−/−^ cells than wild type cells (Fig. 6E). Combined with the poor ability to be transfected with DNAs (Fig. S7B), we concluded that MIM plays a general role in the uptake of extracellular particles

## Discussion

The presented study here supports a role of endogenous MIM in the regulation of the actin cytoskeletal architecture and cell polarity. This function is consistent with the abundant expression of MIM in cerebellar Purkinje cells [1], the largest and extremely polarized neurons replete with extensive dendritic spines, and more recently described functions of MIM in cilla maintenance [22] and kidney epithelial cell contacts [26]. Although the detailed mechanism for MIM to regulate cell architecture remains unclear, previous studies have documented the function of MIM and other I-BAR domain proteins in inducing membrane protrusions. Overexpression of MIM, Abba or IRSp53, in particular the constructs containing their I-BAR domains only, often triggers extensive membrane protrusions morphologically similar to filopodia [12], [27], [28]. However, the ability of MIM to induce membrane protrusions appears to be independent on actin because peptides corresponding to the MIM's I-BAR domain are sufficient to deform synthesized PI(4,5)P_2_-rich membranes into protrusive structures even in the absence of actin [12]. Thus, the MIM-mediated membrane protrusion may represent a cellular structure different from filopodia, which are often manifested as short and straight microprotrusions and assembled by Cdc42-mediated actin polymerization [29]. In agreement with this notion MIM^−/−^ cells have a normal content of filopodia and show no significant changes in Cdc42 activation under the conditions tested. Also, MIM null *Drosophila* cells [30] and murine cells with depletion of IRSp53 [31] exhibit normal filopodia formations, confirming a dispensable function of the I-BAR domain proteins in filopodia formation. Interestingly, the membrane projections, which were diminished in MIM^−/−^ cells, are often enriched with stress fibers. Since MIM is implicated in membrane curvature, it is possible that the MIM-mediated membrane deformation may provide an intracellular compartment to recruit newly assembled actin bundles, which in turn further promote contractions and stabilize membrane projections. In the preparation of this manuscript, Saarikangas et al have also recently described a MIM complete knockout mouse strain [26]. While the focus of the study was on animals other than cells, the authors have found that their MIM knockout mice developed normally and were fertile, the result that was consistent with our finding. They also found that the mice had compromised integrity of kidney epithelia intercellular junctions, supporting a positive role of MIM in actin assembly.

MIM^−/−^ cells also exhibited different migratory ability in a manner depending upon growth conditions. Their mobility was ostensibly impaired in medium deprived of growth factors but appeared to be normal if the medium supplemented with growth factors or serum. Such property may have an important pathological implication because the loss of MIM expression is often associated with certain tumor cells that are more motile or metastatic. Reduced MIM expression in tumor cells may pathologically impinge on the response of these cells to tumor microenvironments by favoring migration toward higher concentrations of growth factors released from other sources where pathogenesis arises, and thereby facilitating tumor cells to travel to distant sites. The established MIM knockout mice should serve as an ideal animal model to test whether depletion of MIM expression would contribute to tumor progression under different pathological conditions.

We also observed that MIM^−/−^ cells were partially impaired in the internalization of PDGF receptors, transferrin, foreign DNAs and chemicals, the finding that agrees with the reported function of other BAR or I-BAR domain proteins in endocytosis [32]–[37]. In particularly, it has been recently reported that knockdown of CLIP4-like proteins, which belong to the F-BAR domain subfamily, has resulted in a prolonged formation of PDGF-induced dorsal ruffles along with delayed endocytosis of PDGF receptor beta [38]. The similar mechanism may also explain the apparently enhanced Rac activity, profound dorsal ruffling and PDGF receptor signaling in MIM deficient cells in response to PDGF where complete endocytosis of PDGF receptors was also partially impaired. MIM may regulate endocytosis through modulation of membrane deformation. It is also possible that the attenuated Rho activity in MIM^−/−^ cells could account for the decrease in internalization of extracellular particles because of a positive role of Rho in some macropinocytoses [39], [40] and typeII phagocytosis [41]. However, our observed positive role of MIM in endocytosis is not in line with a recently reported anti-endocytosis of MIM in MEFs and in *Drosophila* cells [20]. This apparent discrepancy might be explained by different biological systems used in these studies. In particular the study reported by the Oro's group involved Ptc1-null MEFs where MIM was overexpressed.

MIM^−/−^ cells often show higher contents of cortactin-enriched lamellipodia than wild type cells when growing in a regular serum-containing medium. Both lamellipodia and ruffles are similar dynamic structures with ruffles created as a consequence of insufficient adhesions to substratum [42] and are controlled by WAVE-1 and WAVE-2 [43]. Previous studies have indicated that IRSp53 acts as an intermediate between Rac1 and WAVE2 [44], and suppression of IRSp53 expression can diminish the Rac1-mediated surface ruffles [31]. This suggests that MIM may regulate membrane ruffling in a different manner as IRSp53. Instead of promoting ruffles, endogenous MIM could inhibit ruffles, in particularly dorsal ruffles. Consistent with this possibility, MIM^−/−^ cells have higher contents of Rac1 activity than control cells during response to PDGF. It has been recently reported that MIM antagonizes cortactin, which is also a ruffle- and lamellipodia-associated protein that participates in actin polymerization [45]. While we did not observe significant changes in cortactin expression or tyrosine phosphorylation (data not shown) in MIM^−/−^ cells, we could not rule out the possibility that the absence of MIM might release cortactin from the MIM-mediated inhibition, thereby increasing the assembly of cortical actin networks. Defining of the exact mechanism for MIM to regulate small GTPases and its physiological relationship with cortactin would eventually allow us to understand better the function of MIM in the regulation of cell dynamics.

## Materials and Methods

### Ethic Statement

All animal experiments were conducted in compliance with protocol (0806015) instituted by the Institutional Animal Care and Use Committee of the University of Maryland School of Medicine.

### Preparation of the MIM knockout mice

A mouse embryonic stem (ES) cell line (clone CSC156) carrying a gene trapping vector (pGT0Lxf) was obtained from BayGenomics. Genomic PCR and DNA sequencing determined that the vector was inserted at the site of nucleotide 57,067 in the third intron. The trapping vector insertion would predict either a out-frame splicing of the 3rd exon to the beta-gal-neo fusion gene (geo) in the vector or an in-frame splicing between the second exon with the geo gene. In either case, the predicted protein product would contain no more than 70 amino acids in the I-BAR domain. Verified ES cells were microinjected into blastocysts derived from a female C57BL/6 mouse and further transferred into a pseudo-pregnant C57BL/6 mouse. Male chimera offspring of the injected mice was used to generate multiple heterozygous mice, which were further inbred to generate homozygous mice. The genotype of the targeted mice and embryos was determined by genomic PCR. The primers used to determine the wild type allele were 5′-GTAAGGCTGACTGGCGTGGC-3′/5′-ATACGGCTTACCATCATCACACGAC-3′; and the primers used for the mutated allele were 5′-GTAAGGCTGACTGGCGTGGC-3′/5′-TGAGCACCAGAGGACATCCGG-3′. To achieve a homogenous genetic background, targeted mice were backcrossed with C57BL/6 strain for six to nine generations. Mouse embryonic fibroblasts (MEF) were generated from homozygous and wild type E13.5 embryos with otherwise the same genetic background.

### Reagents and antibodies

All chemicals unless otherwise indicated were purchased from Sigma Aldrich. Alexa 568 or 488-conjugated phalloidin were from Invitrogen. Protein A/G plus-agarose was from Santa Cruz. SuperFect was from Qiagen, and Lipofectamine 2000 was from Invitrogen. Antibodies used in this study were from the sources as follows: monoclonal anti-Rac1 (Upstate), polyclonal anti-Cdc42 (Cell Signaling Technology), polyclonal anti-Caveolin (Transduction Laboratory), polyclonal anti-Arp3 (Santa Cruz), polyclonal anti-dynamin (Transduction Laboratory), monoclonal anti-cortactin and monoclonal anti-Rho (A, B and C) (Millipore), monoclonal anti-galactosidase (Promega), monoclonal anti-actin (Sigma), monoclonal anti-alpha-tubulin (Sigma), and monoclonal anti-vinculin (Sigma). Rabbit polyclonal anti-MIM antibody was prepared as previously described [19]. Horseradish peroxidase-conjugated antibodies against goat, mouse and rabbit IgG, fluorescein isothiocyanate and rhodamine-conjugated anti-rabbit IgG antibodies were from Pierce. NSC23766 (Rac1 inhibitor) was purchased from Calbiochem.

### Cell culture

MEFs were prepared as described previously [46], and cultured in Dulbecco's Modified Eagle Medium (DMEM) supplemented with 10% bovine calf serum (Hyclone), 100 U/ml penicillin, 100 µg/ml streptomycin, 2 mM glutamine, 1 mM non-essential amino acids, and 1 mM sodium pyruvate. For the all cellular and biochemical analyses, wild type and MIM^−/−^ MEFs at the similar passages were used and compared.

### Plasmid

Plasmid Myc-RhoA^Q63L^ was purchased from Addgene, Inc.

### Analysis of activation of small GTPases

The Rac1 or Cdc42 activity was measured by pull-down assay. Briefly, cells were cultured in 6-cm dishes to 90% confluence and lysed by addition of 0.5 ml of ice-cold lysis buffer (50 mM Tris-HCl, pH 7.4, 1 mM EDTA, 150 mM NaCl, 1 mM phenyl methylsulphonyl fluoride, 1% Triton-X 100, 1 mM sodium fluoride and Roche proteinase inhibitor cocktail tablet). The lysates were clarified by centrifugation at 16,100× *g* for 5 min at 4°C. The clarified lysates were incubated at 4°C with 30 µl slurry of GST-PAK-CRIB and glutathione Sepharose-4B beads (Amershem Bioscience). After 10 min of incubation, the beads were briefly precipitated and washed three times with lysis buffer. The bound proteins were eluted by boiling in 2× SDS-sample buffer and then subject to 12% SDS-polyacrylmide gel electrophoresis followed by Western blot with Rac1 or Cdc42 antibody. To evaluate the Rho activity, cells were lysed and processed as described above. The GTP bound Rho in the lysates was precipitated with 40 µl of Rhotekin RBD beads (Millipore) and further detected by Western blot using a pan-Rho antibody.

### Morphological analysis

MEFs were seeded onto fibronectin-coated coverslips, cultured overnight and arrested by incubation for 48 h in DMEM containing 0.2% calf serum. Arrested cells were stimulated by adding PDGF at the concentration of 30 ng/ml. After 10 min incubation, cells were fixed with 4% peraformaldehyde and stained with primary antibody followed by staining with FITC-conjugated secondary antibody and Alexa546-conjugated phalloidin. Stained cells were inspected under Nikon TE-2000 U microscope equipped with a UV lamp and digital camera Nikon DXM1200F using either a Plan fluor 20× objective lens or a Plan Apo 60× oil objective with numerical aperture value of 1.40. To quantify membrane ruffling and lamellipodia, the area representing ruffling and lamellipodia on captured images were digitally outlined and measured with MetaMorph software. To quantify filopodia, the number of filopodia protrusions of each cell was counted under microscope. For each sample, at least 70 cells were counted. The difference between wild type and MIM^−/−^ cells was statistically analyzed by Student's *t*-test or ANOVA test when cells were analyzed at multiple time points.

### Cell spreading assay

Cells were trypsinized, seeded on fibronectin-coated coverslips or regular tissue culture dishes in DMEM supplemented with 10% serum, and incubated at 37°C with 5% CO_2_ supply. After attachment, cell images were taken at times from 30 min to 2 h. The attached cells as determined by the appearance of apparent extension of the cytoplasm were countered. To visualize dorsal ruffling during cell attachment, cells were fixed, stained with Alexa488 phalloidin and inspected with laser confocal microscopy as described previously [46].

### Wound healing assay

5×10^5^ cells were seeded onto a 6-cm plate and cultured overnight in DMEM containing 10% serum. When cell cultures reached to confluence, a wound on the monolayer was created by a rubber policeman perpendicular to an ink-marked reference line labeled on the dish. Cells were then incubated in medium with either 0.2% or 10% serum for 22 h. The healing process was digitally recorded, and the reference mark was used to identify the same area for image acquisition at different times. The migration rate was calculated as the ratio of the width of the initial wound to that recorded after 22 h.

### Transwell migration assay

Cells were grown to 70% confluence and arrested in medium containing 0.2% serum for overnight. The arrested cells were trypsinized, resuspended in DMEM and plated on Transwell fibronectin-coated membrane insert of 8 µm pore at a density of 3×10^4^ cells per well. The lower chamber was filled with either 0.2% serum-containing medium or plus PDGF (30 ng/ml) or EGF (10 ng/ml) or 10% calf serum. The plated cells were allowed to migrate for 4 h in a humidified incubator containing 5% CO_2_ at 37°C. The cells remained on the top of the insert were removed with a cotton swab. The cells migrated to the lower surface of the insert were fixed in 4% paraformaldehyde in PBS for 30 min at room temperature, stained with Harris hematoxylin-modified solution for 30 min and viewed under a phase-contrast microscope (200× magnification). Digital images of five random microscopic fields were captured, and the number of migrated cells was counted using MetaMorph 6.1 program.

### Time lapse recording

We used the Axiovert 200 microscope (Zeiss, Germany) equipped with temperature and CO_2_ controlled unit XL-3 and Axiovision software. Cells to be examined were grown in DMEM supplemented with 10% serum in a 6-well plate, and cell images were recorded at interval of 10 seconds for additional 45 min. The recorded digital image stacks were compiled to a movie clip with a frequency of 5 pictures per second using Axiovision software (Zeiss, Germany).

### Endocytosis and micropinocytosis

Cells were seeded in 12-well plates at the concentration of 5×10^4^ cells/well, cultured overnight in DEME containing 10% serum and then starved in DMEM supplemented with 1% bovine serum albumin and 20 mM HEPEs. After incubation at 37°C for 30 min, 10 ug/ml of biotin-labeled transferrin (Bio-Tfn) was added into the medium and the cultures were incubated for 30 min on ice. Endocytosis was initiated by incubating the cell samples at 37°C for 5 min and terminated by placing on ice. The total cell lysates were then transferred to a 96-well ELISA plate coated with anti-Tfn antibody. After 12 h of incubation at 4°C, each well was added with streptavidin-horseradish peroxidase and Roche BM blue substrate, respectively. Absorption at 450 nm was determined by a microplate reader (Anthos Labtec).

For macropinocytosis analysis, cells were seeded in a 12 well plate at density of 4×10^5^ cells/well and then cultured at 37°C with 5% CO_2_ supple overnight. The cells were washed three times with PBS, and incubated with DMEM containing 25 µM Alexa Fluor 647 for 15, 60 and 90 min, respectively. After each time point the cells were trypsinized and then collected for flow cytometric analysis. The intensity of internalized Alexa Fluor 647 in live cells was recorded as the average for 5000 cells in each measurement. Data were processed based on three independent experiments at each time point.

### Internalization of PDGF receptors

Uptake of PDGFR alpha was performed according to the method as described [38] with some minor modifications. Briefly, cells were starved overnight and then stimulated with 50 ng/ml PDGF-BB (Invitrogen) at 37°C for different times. The stimulated cells were washed with cold PBS, and the cell surface receptors were labeled by incubation with 0.2 mg/ml sulfo-N-hydroxysuccinimido (NHS)-SS-biotin (Pierce) in PBS for 1 h on ice. Unbound sulfo-NHS-SS-biotin was inhibited and washed away by 50 mM Tris pH 8.0. After lysis, the labeled proteins were precipitated by incubation with streptavidin-agarose (Sigma) for 1 h at 4°C. The beads were washed three times with lysis buffer and boiled with SDS sample buffer. Eluted proteins were separated by SDS-PAGE (8% v/v) followed by Western blot using PDGFRα antibody. Total amount of cell-associated PDGFRα was measured by Western blot of aliquots of the lysates derived from stimulated cells.

## Supporting Information

Figure S1 **Depletion of MIM expression in Purkinje cells of MIM^−/−^ mice.** Cerebellum sections were stained with MIM antibody. Strong staining was evident in Purkinje cells of the molecular layer (arrow) in wild type but not MIM−/− mice.
(TIF)

Figure S2 **Knockout of MIM does not affect cell proliferation.** MIM^−/−^ and MIM^+/+^ cells were seeded in triplicates in 24-well plates at the density of 2×10^4^ cells/well and incubated in a serum-containing medium. After 48, 96 and 144 h of plating, cells were trypsinized and counted.
(TIF)

Figure S3 **MIM KO cells form poorly focal adhesions (FA).** (A) MEFs were plated on fibronectin-coated cover slips in the presence of serum and stained with vinculin antibody (green) and phalloidin (red). (B) Quantification of the percentage of cells that were devoid of prominent FA as defined by alignment with stress fibers (n = 3).
(TIF)

Figure S4 **MIM deficiency does not alter significantly expression of Rho proteins.** Starved MEFs were treated with PDGF (A) or 10% serum (B) for the times as indicated and then analyzed for the expression of actin and Rho proteins by Western blot using Rho antibody recognizing RhoA, B, and C. ON, overnight incubation.
(TIF)

Figure S5 **Constitutively activated RhoA restored partially stress fiber formation.** (A) MIM−/− MEFs were transiently transfected with Myc-Rho^Q61L^ (a and b), transfection reagents only (c and d), or with GFP-RhoA (e and f). After transfection, cells were plated on coverslips and stained with phalloidin (a, c and e) or Myc antibody (b and d). The stained cells were inspected by epimicrography. A GFP-MIM expressing cell was indicated by * in e and f. Scale bars: 20 µm. (B) Quantification of MIM−/− cells with prominent stress fibers after transfections based on three independent experiments (mean ± SEM).
(TIF)

Figure S6 **MIM^−/−^ cells display a distinct morphology.** A, primary MIM^+/+^ MEFs; B, primary MIM^−/−^ MEFs; C, established WT MEFs; and D, established MIM^−/−^ MEFs. All the cells were grown in serum-containing medium.
(TIF)

Figure S7 **Expression of GFP-MIM restored partially generation of membrane projections in MIM^−/−^ cells.** MEFs were transiently transfected with Myc-MIM (A) or GFP-cortactin constructs (B). After two days of transfection, cells were lysed and analyzed by Western blot using Myc, and GFP antibody, respectively. Note, transfection efficiency of both Myc-MIM and GFP-cortactin in MIM^−/−^ cells was much poorer than that in WT cells. (C), MIM^−/−^ MEFs were transiently transfected with a GFP-MIM or GFP construct. After two days of transfection, the cell cultures were inspected by fluorescent (left) or phase-contrast (right) microscopy. A transfected GFP-MIM cell and a GFP cell were indicated by *.
(TIF)

Figure S8 **MIM is not required for the formation of filopodia.** MIM KO (A) and WT (B) cells were stimulated with PDGF for 10 min and stained with phalloidin. Arrow heads indicate some representative filopodia. (C) Quantification of the numbers of filopodia per cell. (D) GTP-Cdc42 was measured by pull-down using GST-PAK-GRIB beads followed by Western blot with Cdc42 antibody. Quantification of Cdc42 activation based on six experiments (mean ± SEM) is presented at bottom.
(TIF)

Figure S9 **MIM−/− cells have higher contents of activated Rac1 protein upon PDGF treatment.** Starved MEFs were treated with PDGF (30 ng/ml) for the times as indicated. GTP-Rac1 was measured by pull-down assay. As controls, cells maintained in 10% serum-containing medium (S) were also analyzed in parallel.
(TIF)

Video S1 **MIM^−/−^ cells are more dynamic in cell leading edges.** WT cells were grown in a 6-well plate in a medium containing 10% serum. Time-lapse imaging was performed on Zeiss Axiovert 200 microscope equipped with environment control chamber at a rate of 12 frames per minute for total 45 min as described in the Materials and Methods.
(WMV)

Video S2 **MIM^−/−^ cells are more dynamic in cell leading edges.** MIM^−/−^ cells were grown in a 6-well plate in a medium containing 10% serum. Time-lapse imaging was performed on Zeiss Axiovert 200 microscope equipped with environment control chamber at a rate of 12 frames per minute for total 45 min as described in the Materials and Methods. The arrows indicate two dynamic dorsal ruffles in MIM KO cells.
(WMV)
