# Supplementary figures and images for: Murine Missing in Metastasis (MIM) Mediates Cell Polarity and Regulates the Motility Response to Growth Factors
Source: PLoS One. 2011 Jun 9;6(6):e20845. doi: 10.1371/journal.pone.0020845 (PMC3111439; doi:10.1371/journal.pone.0020845)

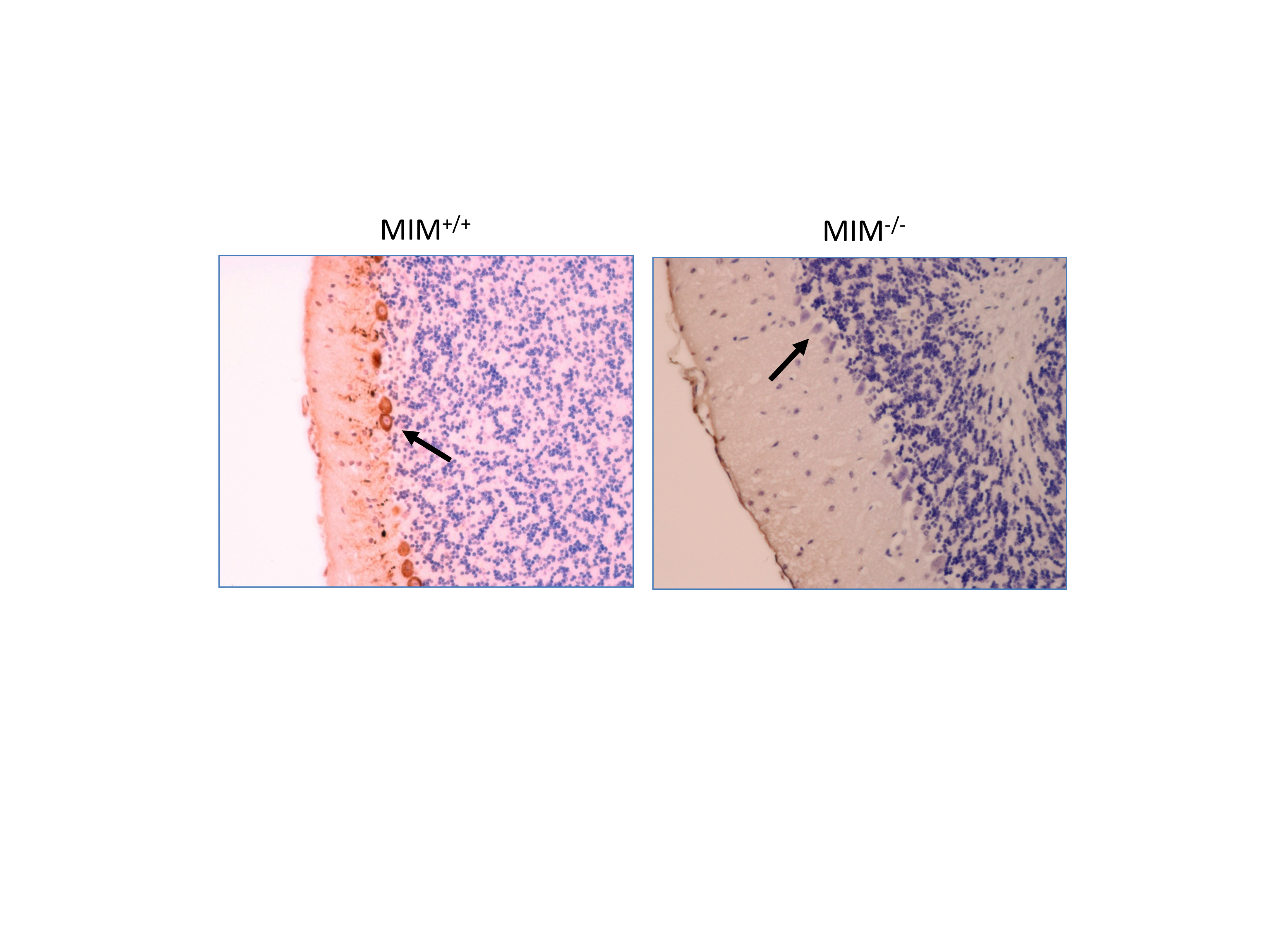

Supplement: Figure S1 — Depletion of MIM expression in Purkinje cells of MIM−/− mice. Cerebellum sections were stained with MIM antibody. Strong staining was evident in Purkinje cells of the molecular layer (arrow) in wild type but not MIM−/− mice. (TIF) [file pone.0020845.s001.tif]

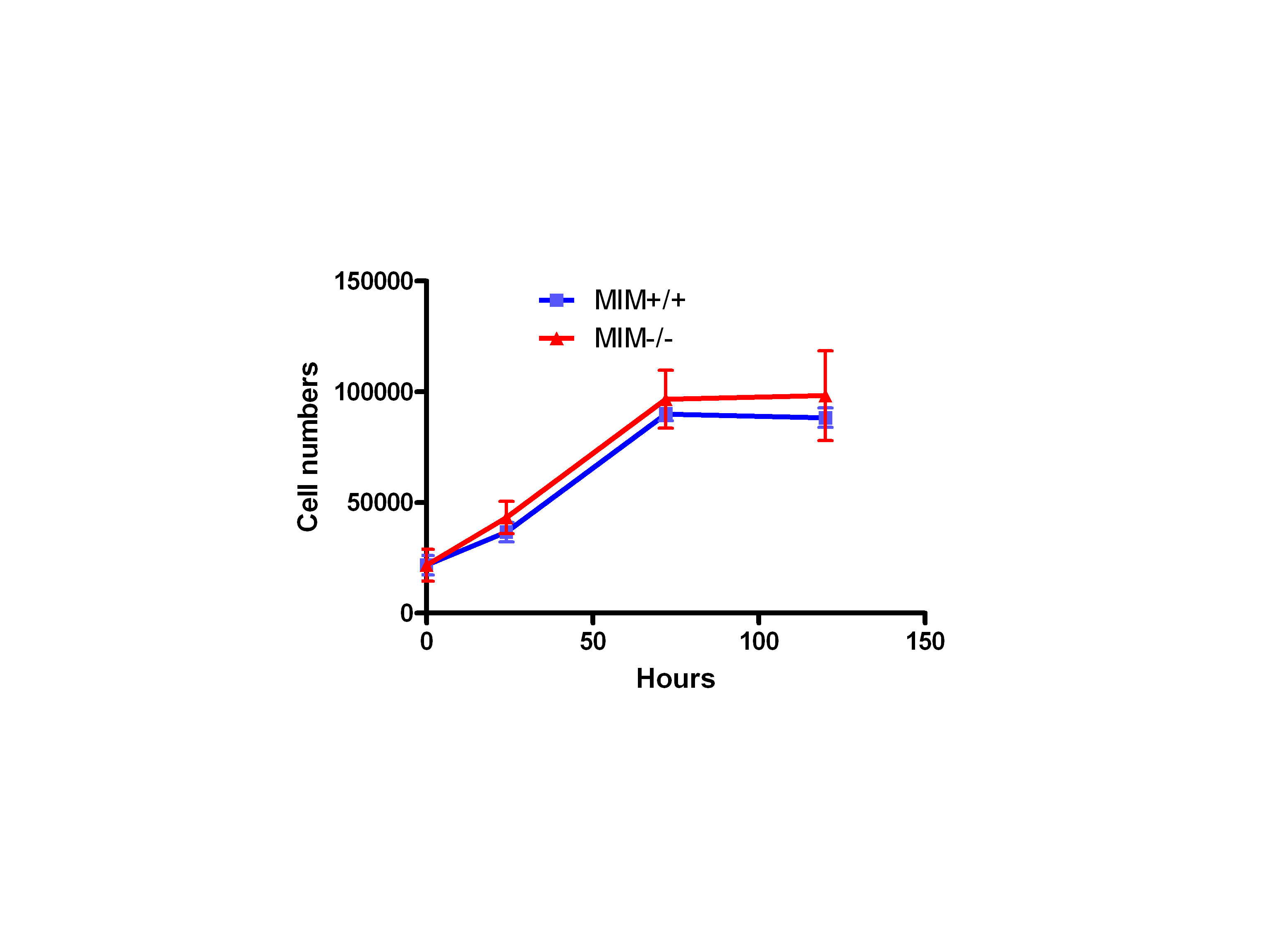

Supplement: Figure S2 — Knockout of MIM does not affect cell proliferation. MIM−/− and MIM+/+ cells were seeded in triplicates in 24-well plates at the density of 2×10^4^ cells/well and incubated in a serum-containing medium. After 48, 96 and 144 h of plating, cells were trypsinized and counted. (TIF) [file pone.0020845.s002.tif]

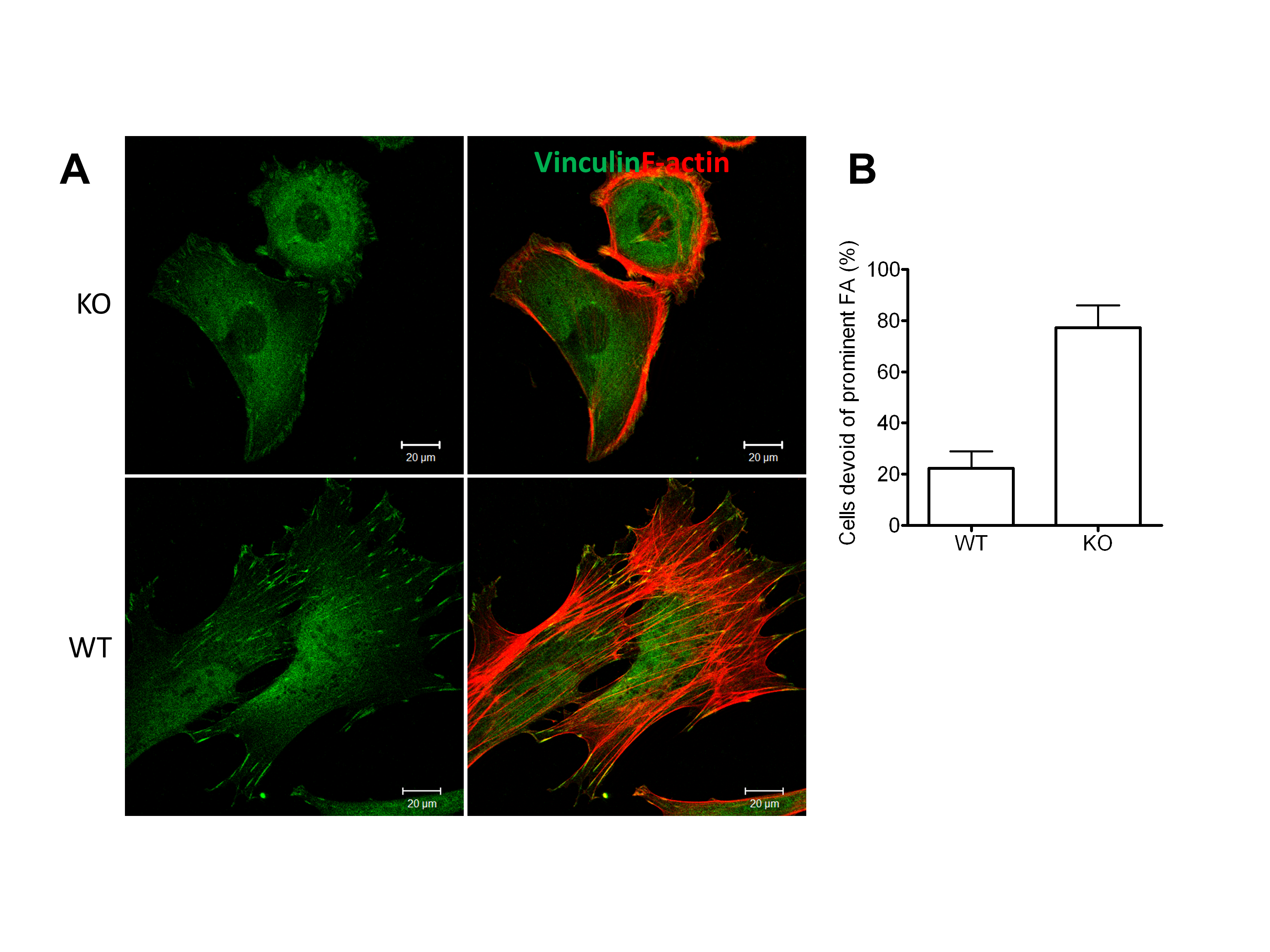

Supplement: Figure S3 — MIM KO cells form poorly focal adhesions (FA). (A) MEFs were plated on fibronectin-coated cover slips in the presence of serum and stained with vinculin antibody (green) and phalloidin (red). (B) Quantification of the percentage of cells that were devoid of prominent FA as defined by alignment with stress fibers (n = 3). (TIF) [file pone.0020845.s003.tif]

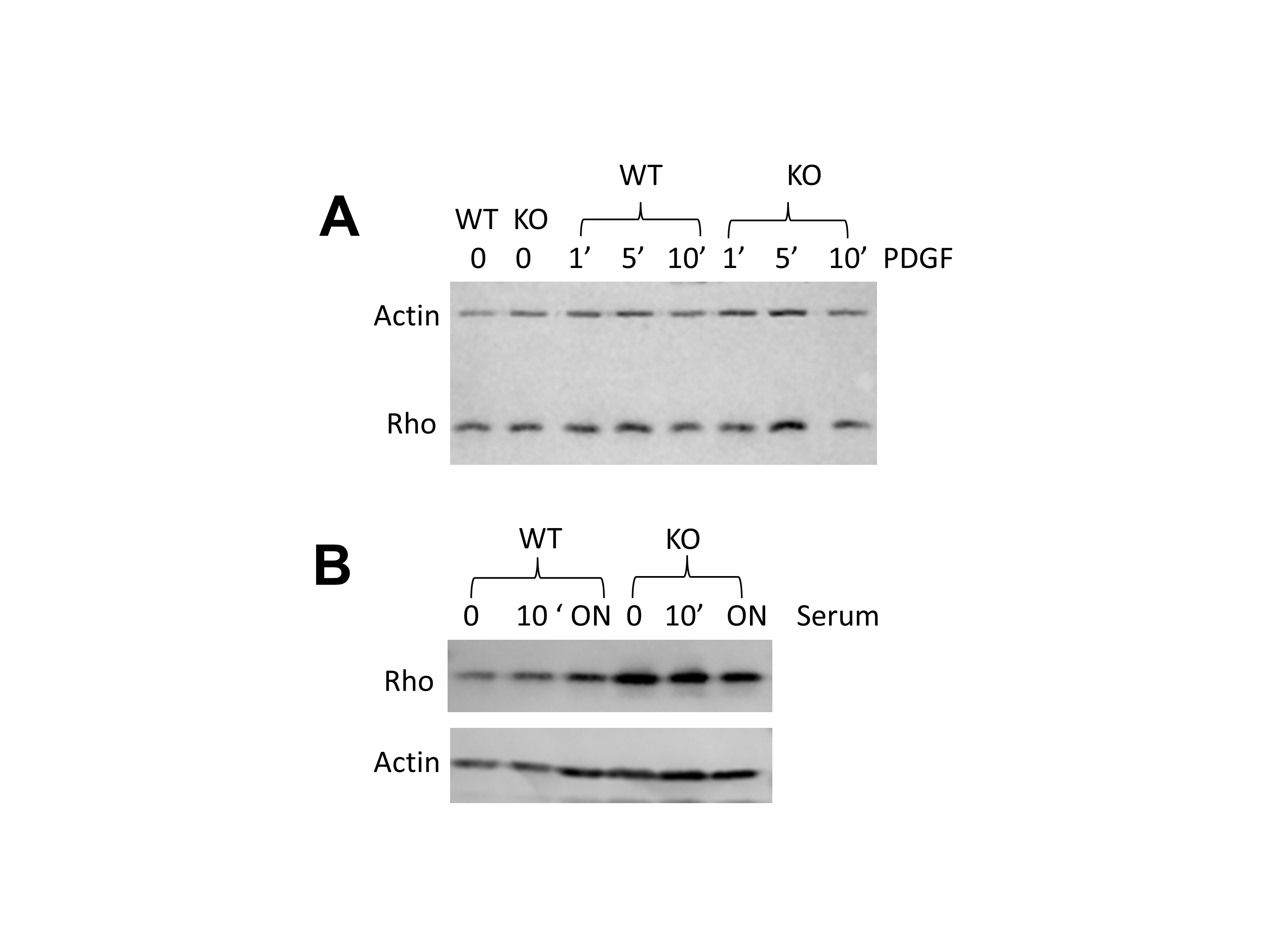

Supplement: Figure S4 — MIM deficiency does not alter significantly expression of Rho proteins. Starved MEFs were treated with PDGF (A) or 10% serum (B) for the times as indicated and then analyzed for the expression of actin and Rho proteins by Western blot using Rho antibody recognizing RhoA, B, and C. ON, overnight incubation. (TIF) [file pone.0020845.s004.tif]

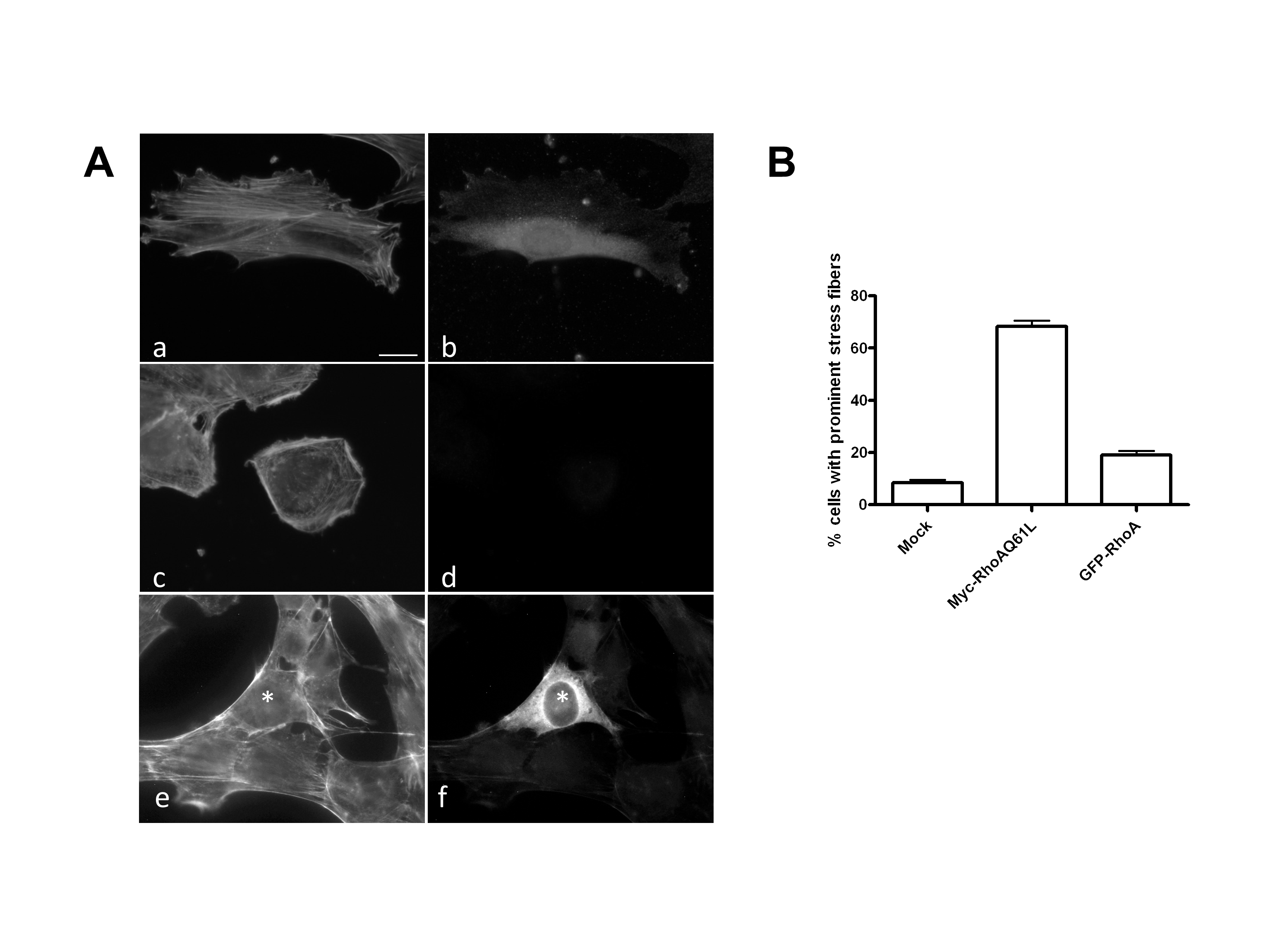

Supplement: Figure S5 — Constitutively activated RhoA restored partially stress fiber formation. (A) MIM−/− MEFs were transiently transfected with Myc-RhoQ61L (a and b), transfection reagents only (c and d), or with GFP-RhoA (e and f). After transfection, cells were plated on coverslips and stained with phalloidin (a, c and e) or Myc antibody (b and d). The stained cells were inspected by epimicrography. A GFP-MIM expressing cell was indicated by * in e and f. Scale bars: 20 µm. (B) Quantification of MIM−/− cells with prominent stress fibers after transfections based on three independent experiments (mean ± SEM). (TIF) [file pone.0020845.s005.tif]

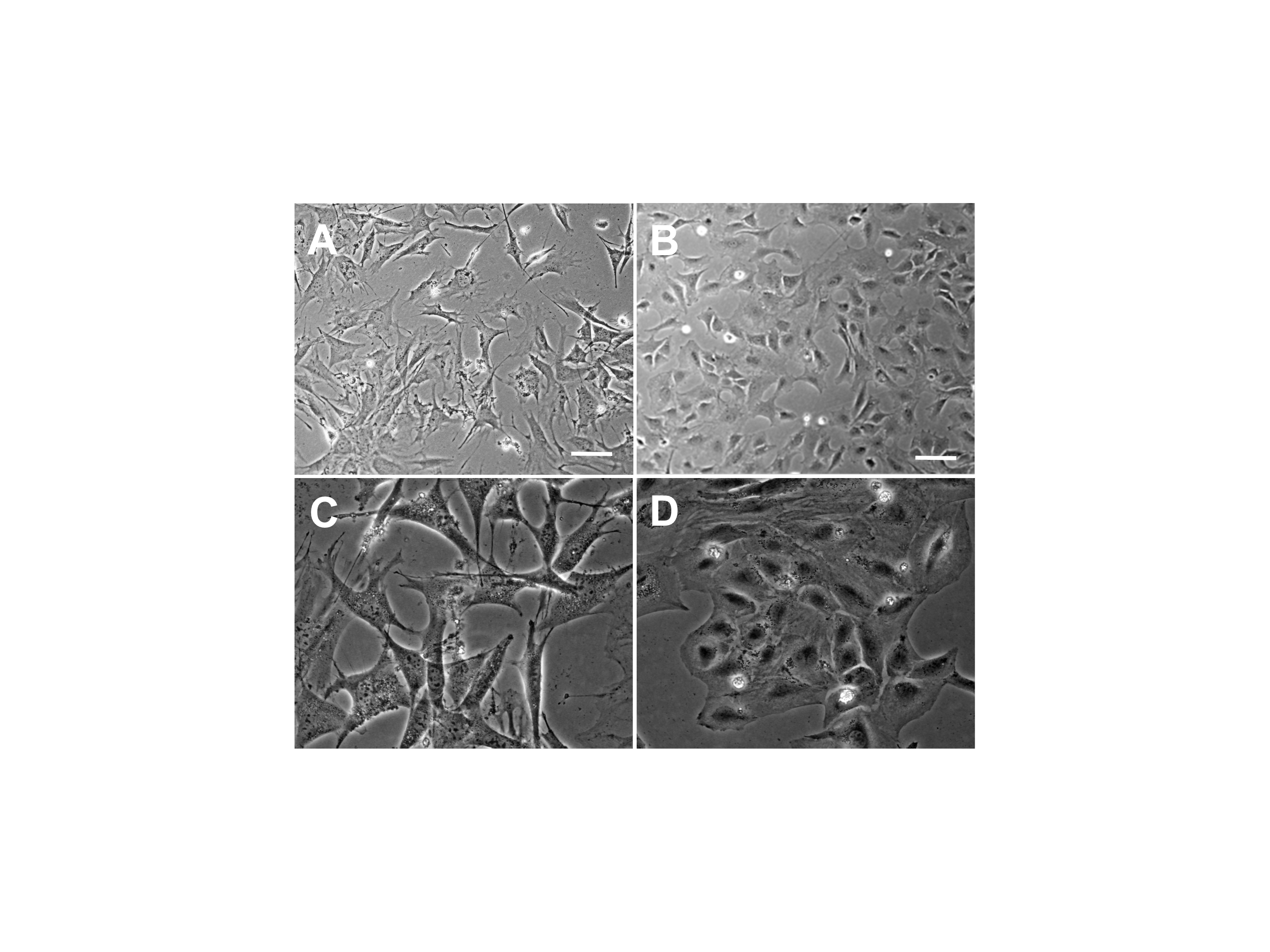

Supplement: Figure S6 — MIM−/− cells display a distinct morphology. A, primary MIM+/+ MEFs; B, primary MIM−/− MEFs; C, established WT MEFs; and D, established MIM−/− MEFs. All the cells were grown in serum-containing medium. (TIF) [file pone.0020845.s006.tif]

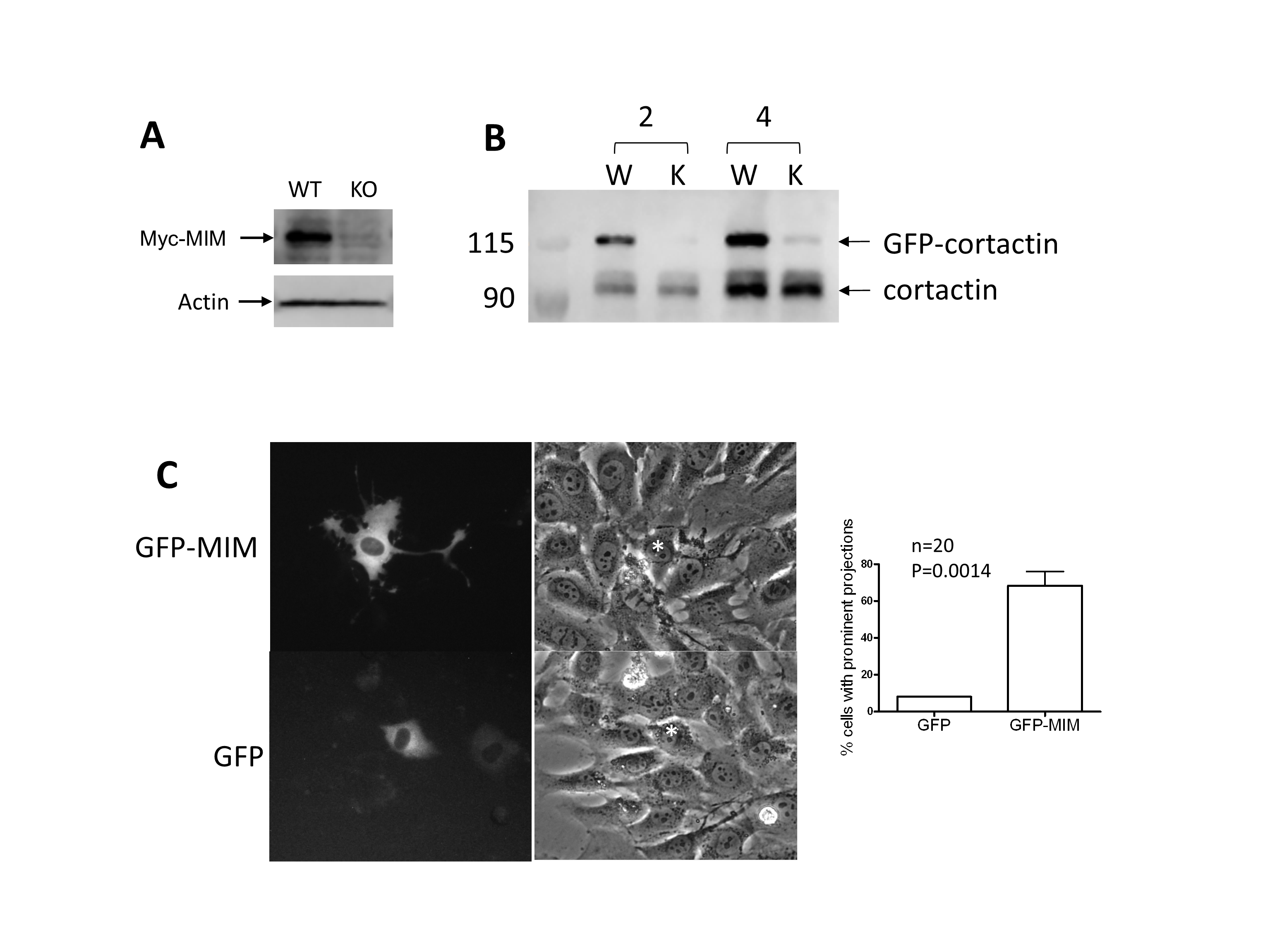

Supplement: Figure S7 — Expression of GFP-MIM restored partially generation of membrane projections in MIM−/− cells. MEFs were transiently transfected with Myc-MIM (A) or GFP-cortactin constructs (B). After two days of transfection, cells were lysed and analyzed by Western blot using Myc, and GFP antibody, respectively. Note, transfection efficiency of both Myc-MIM and GFP-cortactin in MIM−/− cells was much poorer than that in WT cells. (C), MIM−/− MEFs were transiently transfected with a GFP-MIM or GFP construct. After two days of transfection, the cell cultures were inspected by fluorescent (left) or phase-contrast (right) microscopy. A transfected GFP-MIM cell and a GFP cell were indicated by *. (TIF) [file pone.0020845.s007.tif]

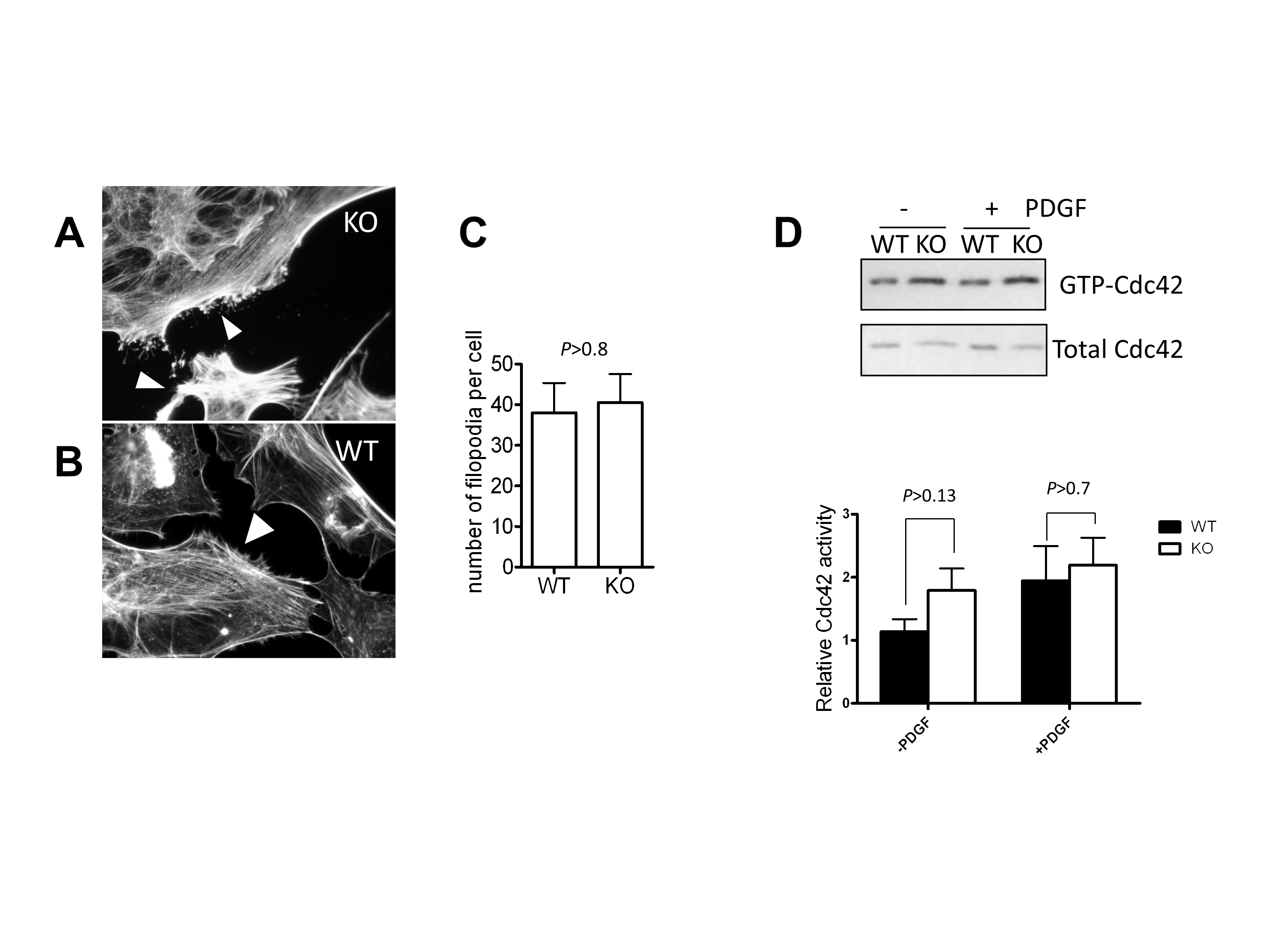

Supplement: Figure S8 — MIM is not required for the formation of filopodia. MIM KO (A) and WT (B) cells were stimulated with PDGF for 10 min and stained with phalloidin. Arrow heads indicate some representative filopodia. (C) Quantification of the numbers of filopodia per cell. (D) GTP-Cdc42 was measured by pull-down using GST-PAK-GRIB beads followed by Western blot with Cdc42 antibody. Quantification of Cdc42 activation based on six experiments (mean ± SEM) is presented at bottom. (TIF) [file pone.0020845.s008.tif]

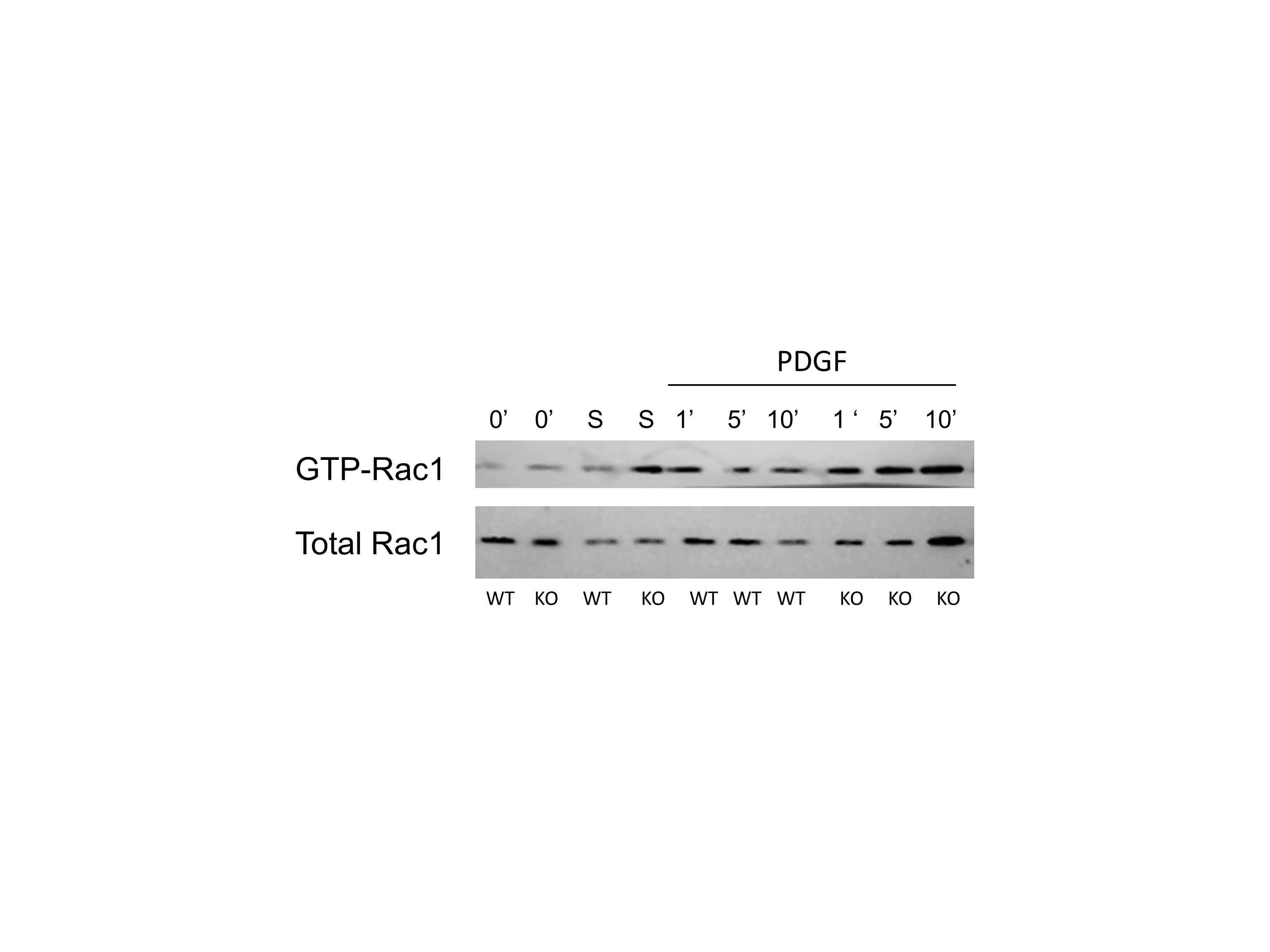

Supplement: Figure S9 — MIM−/− cells have higher contents of activated Rac1 protein upon PDGF treatment. Starved MEFs were treated with PDGF (30 ng/ml) for the times as indicated. GTP-Rac1 was measured by pull-down assay. As controls, cells maintained in 10% serum-containing medium (S) were also analyzed in parallel. (TIF) [file pone.0020845.s009.tif]
